# Green Tea Intake and Parkinson's Disease Progression: A Mendelian Randomization Study

**DOI:** 10.3389/fnut.2022.848223

**Published:** 2022-05-26

**Authors:** Chunyu Li, Junyu Lin, Tianmi Yang, Huifang Shang

**Affiliations:** Laboratory of Neurodegenerative Disorders, Department of Neurology, National Clinical Research Center for Geriatrics, West China Hospital, Sichuan University, Chengdu, China

**Keywords:** green tea intake, Parkinson's disease, Mendelian randomization, age at onset, progression

## Abstract

Epidemiological studies have suggested green tea intake was associated with a reduced risk of Parkinson's disease (PD). However, whether green tea intake has an effect on PD progression is unknown. To evaluate the role of green tea intake in PD progression, we conducted a two-sample Mendelian randomization analysis using summary statistics from genome-wide association studies of green tea intake (*N* = 64,949), age at onset (*N* = 28,568) and progression (*N* = 4,093) of PD. One standard deviation increase in genetically determined green tea intake was significantly associated with slower progression to dementia (OR: 0.87, 95% CI: 0.81–0.94, *P*: 3.48E−04) after the Bonferroni correction. Meanwhile, higher green tea intake was nominally associated with slower progression to depression, and lower risk of dementia, depression, hyposmia and insomnia at baseline. The results were robust under all sensitivity analyses. These results might facilitate novel therapeutic targets to slow down the progression of PD in clinical trials, and have clinical implications for patients with PD.

## Introduction

Parkinson's disease (PD) is a complex progressive neurodegenerative disorder with a wide range of phenotypes like motor, cognitive and affective manifestations (1, 2), which is further entangled by heterogeneous age at onset (AAO) and symptom progression between individuals (3). The interplay of multiple factors like aging, genetics and environmental factors might contribute to such heterogeneity (4). Identifying risk factors for PD progression could help better understand the pathogenesis of the disease, and provide care and therapeutic strategies for patients and clinicians.

Green tea, a widely-consumed beverage, was suggested to have health benefits against cancer, cardiovascular diseases, diabetes and several neurodegenerative disorders including PD (5). Previous case-control designed studies have identified an inverse association between tea intake and risk of PD (6, 7). Pathologically, several potential mechanisms for the protective role of green tea against PD have also been proposed, like the antioxidant potential of green tea in combating oxidative stress and alleviating mitochondrial dysfunction (8). As for the progression of PD, a previous study among 278 patients with PD found that consumption of tea more than 3 cups per day could delay the onset age of motor symptoms (9). However, a recent prospective cohort study investigating lifestyle factors and the progression of PD found that caffeinated tea was associated with reduced mortality but not progression (10). Similarly, a systematic review of prospective longitudinal studies found evidence for a protective role of tea intake against the risk for PD, but not progression of PD (11). Therefore, whether green tea intake has a beneficial role in the progression of PD is still unknown. Meanwhile, the observational studies might be biased by unavoidable confounding factors and relatively small sample size, and cannot determine causation.

In this context, we performed a two-sample Mendelian randomization (MR) analysis to explore the causal role of green tea intake in the progression of PD (Supplementary Figure 1). The MR approach is not susceptible to reverse causation or confounding factors which may distort interpretations of conventional observational studies. As a result, we found that green tea intake was causally associated with a lower risk of dementia in PD.

## Methods

### Datasets

We obtained summary statistics of green tea intake from a genome-wide association study (GWAS) based on the UK Biobank data (*N* = 64,949). Green tea intake was measured by “How many cups/mugs of green tea did you drink yesterday?” in the UK Biobank (data field: 100420). The summary statistics were generated from regression on green tea intake adjusting for sex and principal components after excluding poor quality samples. The detailed design like quality control procedures and statistical analyses could be found at http://www.nealelab.is/uk-biobank/. Single nucleotide polymorphisms (SNP) that passed the genome-wide significance threshold (*P* < 5E−08) were chosen as instrumental variables, which were then clumped based on the 1,000 Genomes Project linkage disequilibrium (LD) structure. Index SNPs (R^2^ < 0.001 with any other associated SNP within 10 Mb) with the minimum *P* value were kept. Furthermore, we used the PhenoScanner v2 tool to check for variants associated with other phenotypes (*P* < 5E−08) which might affect the progression of PD independent of green tea intake (12).

Summary statistics of AAO of PD were from GWAS based on 28,568 PD patients of European ancestry (13). For the progression of PD, we obtained summary data from a large GWAS on clinical biomarkers of PD such as the Hoehn-Yahr (HY) stage in 12 longitudinal PD cohorts (*N* = 4,093) (14). A total of 28 clinical progression phenotypes were analyzed. Harmonization was undertaken to rule out strand mismatches and ensure alignment of SNP effect sizes.

### Mendelian Randomization Analysis

We hypothesized that green tea intake as a protective factor could causally decrease the risk of specific symptom progression in PD, and the following assumptions were satisfied: the genetic variants used as instrumental variables are associated with green tea intake; the genetic variants are not associated with confounders; the genetic variants are associated with PD progression through green tea intake (namely horizontal pleiotropy should not be present) (Supplementary Figure 2).

To evaluate the causative effect of green tea intake on the progression of PD, we performed a two-sample MR analysis using the random effects inverse variance weighted (IVW) method, which is most widely used in MR studies and could provide robust causal estimates under the absence of directional pleiotropy. A *P* value below 1.79E−03 (0.05/28) was considered statistically significant after the Bonferroni correction. For the significant association, we further verified the results using another three MR methods, namely MR Egger regression, weighted median and weighted mode. In addition, we conducted comprehensive sensitivity analyses to estimate potential violation of the model assumptions in the MR analysis. We conducted Mendelian randomization pleiotropy residual sum and outlier (MR-PRESSO) analysis and leave-one-out analysis to detect outlier instrumental variables (15). Outlier instrumental variables identified by the MR-PRESSO analysis were removed step-by-step to reduce the effect of horizontal pleiotropy. Cochran's Q test was executed to check heterogeneity across the individual causal effects. MR-Egger regression was performed to evaluate the pleiotropy of instrumental variables (16). To evaluate the strongness of each instrumental variable, we computed the F-statistic of each SNP as described earlier (17). The statistical analyses were conducted using the R package TwoSampleMR 0.5.5 (18).

## Results

**Figure 1:**
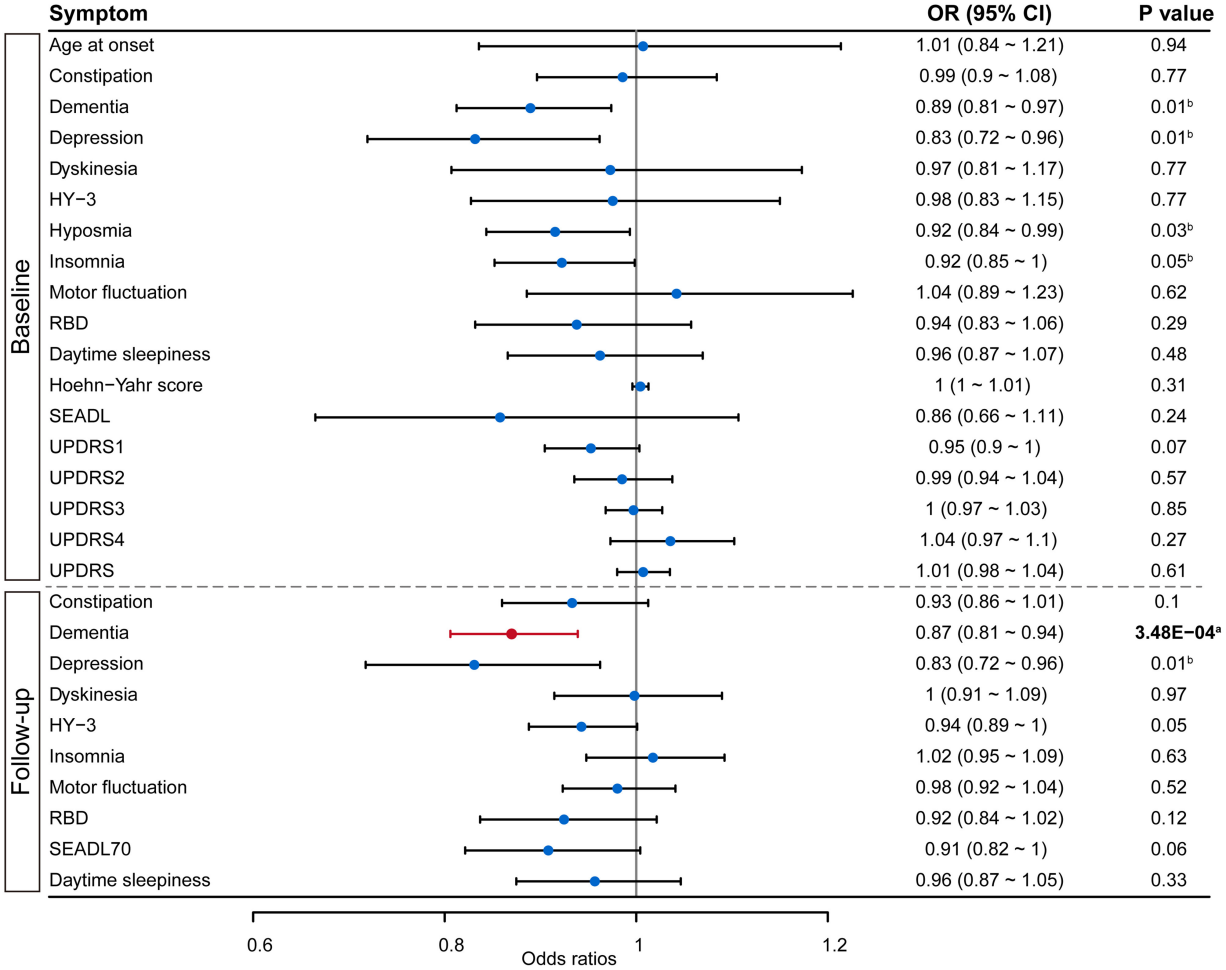
Forest plot showing Mendelian randomization analysis results to evaluate the causal association between green tea intake and progression of PD. Results above the dashed line are for symptoms collected at baseline, while results below the dashed line are from survival analysis on symptom progression during follow-up. SEADL, Schwab and England Activities of Daily Living Scale; UPDRS, Unified Parkinson Disease Rating Scale; HY-3, Hoehn Yahr scale 3 or greater; RBD, REM sleep behavior disorder; SEADL70, SEADL of 70 or less. ^a^Denotes statistical significance, while ^b^denotes nominal significance.

**Figure 2:**
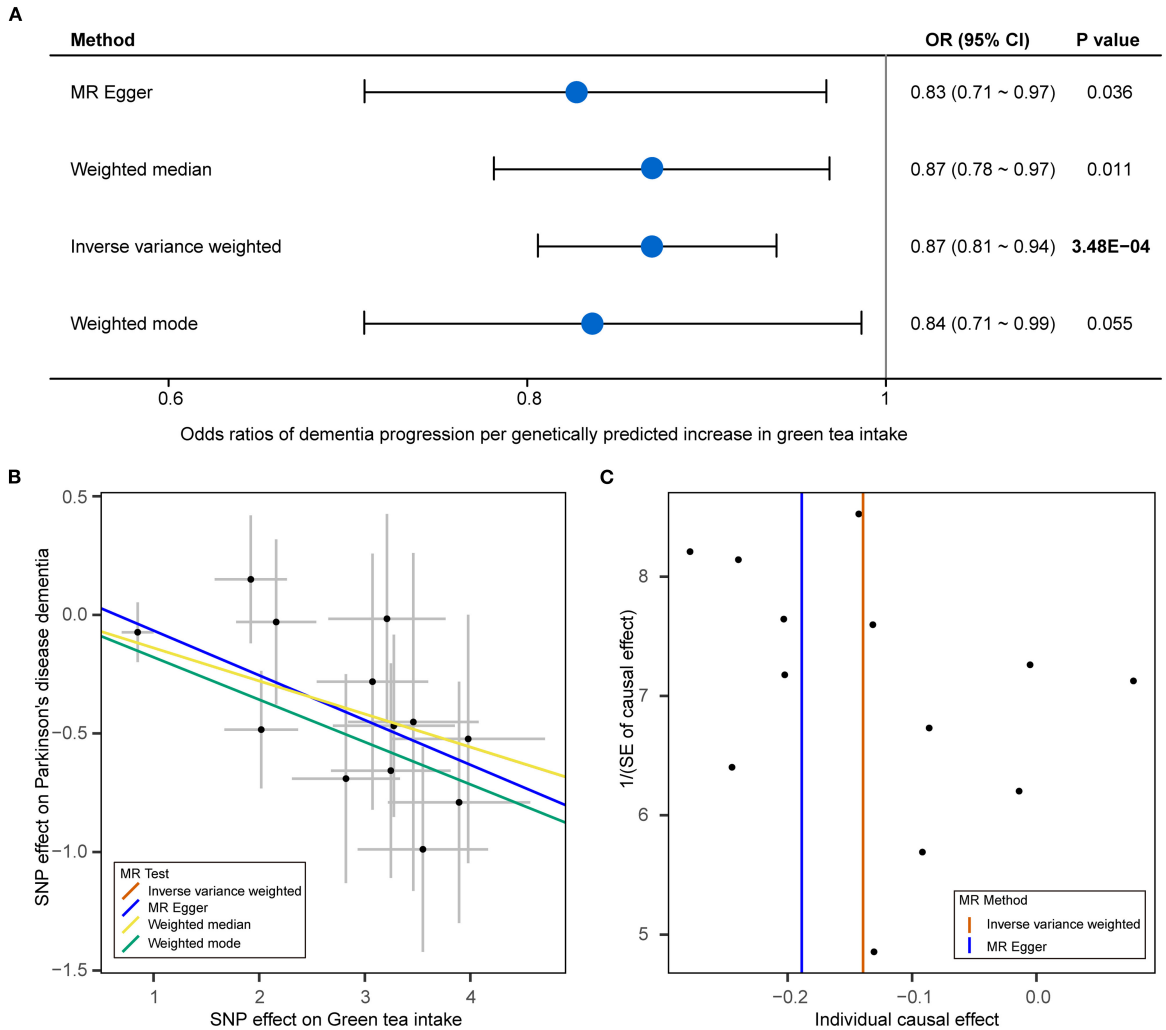
Mendelian randomization analysis results for green tea intake and dementia of PD. **(A)** Forest plot showing Mendelian randomization (MR) analysis results to evaluate the causal association between green tea intake and progression to dementia using four methods. **(B)** Scatter plot of single nucleotide polymorphism (SNP) potential effects on green tea intake and dementia of PD. The 95% CI for the effect size on green tea intake is shown as vertical lines, while the 95% CI for the effect size on dementia of PD is shown as horizontal lines. The slope of fitted lines represents the estimated MR effect per method. **(C)** Funnel plot for green tea intake shows the estimation using the inverse of the standard error of the causal estimate with each individual SNP as a tool. The vertical line represents the estimated causal effect obtained using IVW and MR-Egger methods.

We analyzed the role of green tea intake in the progression of PD using the two-sample MR approach. Results showed that each standard deviation increase in green tea intake was significantly associated with slower progression to dementia (OR: 0.87, 95% CI: 0.81–0.94, *P*: 3.48E−04) after the Bonferroni correction (Figure 1). Such association was further verified using the other three MR methods (Figure 2). Consistently, green tea intake was nominally associated with a lower risk of dementia at baseline, suggesting a protective role of green tea against cognitive impairment in PD. In addition, nominal association was observed between green tea intake and slower progression to depression, and lower risk of depression, hyposmia and insomnia at baseline (Figure 1).

Next, we performed sensitivity analyses to validate the causal association between green tea intake and the progression of PD. No heterogeneity of effects was detected by Cochran's Q test (Table 1). The F statistics of all the instrumental variables were above 10 (ranging from 30 to 44), indicating absence of weakness in the selected instrumental variables. No apparent horizontal pleiotropy was observed as the intercept of MR-Egger was not significantly deviated from zero (Table 1). Meanwhile, no potential instrumental outlier was detected at the nominal significance level of 0.05 by the MR-PRESSO analysis (Table 1). The leave-one-out results suggested that the causal effect was not driven by a single instrumental variable (Supplementary Figures 3–7). Lastly, we used the PhenoScanner tool to check if the SNPs used in the MR analysis were associated with other phenotypes. As a result, no instrumental variable was associated with other phenotypes which might affect the progression of PD independent of green tea intake (Supplementary Table 1).

**Table 1:** Heterogeneity and horizontal pleiotropy analyses between green tea intake and PD progression.

**Trait**	**Heterogeneity**			**Horizontal pleiotropy**			**MR-PRESSO**
	**IVW Q**	**IVW Q df**	**IVW *P* value**	**Egger intercept**	**SE**	***P* value**	***P* value**
Constipation	8.51	12	0.74	0.12	0.2	0.55	0.78
Dementia	7.96	17	0.97	0.13	0.2	0.51	0.96
Depression	4.38	10	0.93	−0.62	0.81	0.47	0.92
Dyskinesia	7.2	10	0.71	0.32	0.42	0.47	0.7
HY-3	9.28	10	0.51	0.70	0.33	0.06	0.49
Hyposmia	6.33	14	0.96	0.13	0.18	0.48	0.97
Insomnia	7.26	14	0.92	0.09	0.17	0.63	0.94
Motor fluctuation	8.46	10	0.58	−0.07	0.36	0.86	0.56
RBD	11.06	12	0.52	0.31	0.28	0.29	0.53
Daytime sleepiness	10.11	10	0.43	0.48	0.61	0.45	0.45
Age at onset	2.63	4	0.62	0.03	0.32	0.92	0.64
HY	8.93	19	0.98	−0.02	0.02	0.26	0.98
SEADL	18.17	16	0.31	0.69	0.6	0.27	0.33
UPDRS1	15.04	12	0.24	2.75E−03	0.12	0.98	0.28
UPDRS2	13.95	12	0.3	−0.24	0.11	0.05	0.3
UPDRS3	12.23	16	0.73	−0.06	0.07	0.38	0.79
UPDRS4	11.01	9	0.28	0.1	0.15	0.51	0.3
UPDRS	9	17	0.94	−0.07	0.06	0.27	0.94
Constipation	8.87	8	0.35	0.25	0.16	0.17	0.34
Dementia	10.72	13	0.63	0.12	0.17	0.51	0.67
Depression	2.61	6	0.86	0.11	0.27	0.7	0.88
Dyskinesia	5.72	11	0.89	0.01	0.18	0.97	0.9
HY-3	13.71	17	0.69	−0.002	0.12	0.99	0.67
Insomnia	9.19	13	0.76	0.13	0.15	0.4	0.76
Motor fluctuation	10.98	14	0.69	0.01	0.13	0.92	70
RBD	14.12	13	0.37	0.54	0.24	0.04	0.36
SEADL70	7.45	14	0.92	−0.07	0.23	0.76	0.94
Daytime sleepiness	15.15	12	0.23	−0.06	0.2	0.78	0.17

*IVW, Inverse variance weighted; Q, Cochran's Q test estimate; df, Cochran's Q test degrees of freedom; SE, standard error*.

*Results above the dashed line were for symptoms collected at baseline, while results below the dashed line were from survival analysis on symptom progression during follow-up*.

## Discussion

Previous epidemiological studies have suggested green tea intake has a protective effect against risk of PD, but whether it could slow down the progression of PD is less explored. Meanwhile, unmeasured confounding factors in clinical studies might potentially bias the association evidence, as is a common criticism inherent to observational studies. Therefore, we investigated the role of green tea intake in the progression of PD using the MR approach. The results demonstrated a protective role of great tea intake against the risk of dementia and depression in PD. Meanwhile, green tea intake was nominally associated with a lower risk of hyposmia and insomnia for patients with PD at baseline. These findings provided a better understanding of the role of green tea intake in the progression of PD, and provided novel targets to explore the pathogenesis of PD.

Previous observational studies have suggested that green tea intake might reduce the risk of dementia, Alzheimer's disease, and cognitive impairment in the elderly (19). However, whether the effect applies to dementia in other disorders was less explored. From a genetic perspective, our results demonstrated the protective role of green tea intake applied to dementia in PD as well, suggesting a universal favorable role of green tea against cognitive impairment. Though how green tea exerts this effect is still unknown, several potential mechanisms have been proposed (19). The first mechanism is the antioxidant activity of green tea catechins in the brain. Oxidative stress has been demonstrated to be involved in the pathogenesis of dementia, while catechins in green tea could chelate bivalent metal ions and prevent oxidation caused by reactive hydroxyl radicals (20). Secondly, several brain inflammatory markers are associated with the risk of all-cause dementia (21), while the anti-inflammatory effects of green tea polyphenols through the inhibition of nuclear factor kappa-beta activation could reduce brain inflammation. Thirdly, epigallocatechin gallate (EGCG), the main component of green tea catechins, has neuroprotective effects due to its inhibition of amyloid-beta aggregation which is closely related to the pathogenesis of dementia (22). Meanwhile, *in vitro* experiments suggested that green tea polyphenols (GTP) could protect dopamine neurons and thus might be beneficial to cognitive function (23). And EGCG was suggested to reduce neuronal cell death and induce nitric oxide synthase (NOS) expression in an MPTP mouse model of PD, which provided further evidence for the neuroprotective role of green tea (24). Additionally, our results also suggested a nominal protective role of green tea intake against depression of PD. Depression is closely related to dementia, and depressive symptoms are considered as the first glimpse of the brain failure that will lead to dementia. The role of green tea against depression in the elderly has been reported by previous observational studies (25). Our results further strengthened this protective effect and broadened the spectrum of this effect to PD. Though the exact mechanism remains elusive, the main components of green tea, namely L-theanine and EGCG might play a role (26, 27). Future clinical or functional studies could attach importance to this in exploring how green tea intake protects against dementia and depression in PD.

Besides, we noticed a nominal protective role of green tea against hyposmia and insomnia at baseline. A previous cohort study found that lower lifetime caffeine consumption was associated with abnormal olfaction in relatives of patients with PD (28). Since caffeine was a major component of green tea, green tea might protect against hyposmia as well. Additionally, daily ingestion of low-caffeine green tea might be beneficial for improving the sleep quality of the elderly via the suppression of stress (29). However, contradictory results have also been reported. For example, one study found that caffeine administration had little or no effect on hyposmia in a group of 76 hyposmic individuals (30). Meanwhile, the caffeine within green tea might also increase the risk of insomnia. Since only nominal association was detected in the current study, further exploration was still necessary to explore the role of green tea intake in hyposmia and insomnia.

In addition, we also noticed an association with borderline significance between green tea intake and progression to HY-3 stage (OR = 0.94, 95 % CI: 0.89–1.00, *P* = 0.054), which described the overall motor symptom progression of PD. Green tea might exert this effect by modulating microglia activation and decreasing the production of inflammatory mediators (31). Similarly, a previous clinical study found that drinking green tea could improve antioxidant status and reduce oxidative damage (32), suggesting the potential beneficial role of green tea in the progression of PD. Pathologically, a previous study has shown that EGCG strongly inhibited the aggregation of α-synuclein and prevented toxicity in PC12 cells (33). Meanwhile, EGCG could also bind to the native unfolded α-synuclein polypeptide chain and prevent the formation of toxic β structures by mediating the formation of the unstructured oligomer. Previous study has shown that EGCG could reduce interaction between α-synuclein oligomers and cell membranes and thus protect rat neuronal cells from toxicity (34). However, a previous epidemiological study did not find an association between tea intake and the time to reach the HY-3 stage of PD (35). Therefore, more evidence was necessary to better understand whether and how green tea was involved in the overall progression of PD.

In contrast, for other symptoms like the Unified Parkinson's Disease Rating Scale (UPDRS) which was shown to benefit from green tea intake, we did not identify a significant association in the current study. This might be due to the different pathogenesis of various symptoms in PD. However, we cannot exclude the possibility that we failed to detect the association due to the insufficiency of the sample sizes. The moderate variance explained by the instrumental variables of the exposure limited the power to detect weaker causal associations. Further validations in larger cohorts were still necessary.

In conclusion, based on results from the MR analysis, we demonstrated a protective effect of green tea intake against dementia and depression of PD. These results could help better understand the role of green tea in the progression of PD, will facilitate therapeutic drugs in the future clinical trials, and also provide some lifestyle recommendation for the patients with PD.

## Data Availability Statement

The original contributions presented in the study are included in the article/Supplementary Material, further inquiries can be directed to the corresponding author.

## Author Contributions

CL: research project—conception and execution, statistical analysis—design, execution, review and critique, and manuscript—writing of the first draft. JL: statistical analysis—execution, manuscript—writing of the first draft, and review and critique. TY: manuscript—review and critique. HS: research project—organization, statistical analysis—review and critique, and manuscript—review and critique. All authors contributed to the article and approved the submitted version.

## Funding

This research was supported by the funding of the National Key Research and Development Program of China (Grant Nos. 2021YFC2501203, and 2021YFC2501205), the Sichuan Science and Technology Program (Grant Nos. 2022ZDZX0023 and 2021YJ0415), and the National Natural Science Foundation of China (Grant Nos. 81901294 and 81871000). The funders had no role in the design and conduct of the study; collection, management, analysis, and interpretation of the data; preparation, review, or approval of the manuscript; and decision to submit the manuscript.

## Conflict of Interest

The authors declare that the research was conducted in the absence of any commercial or financial relationships that could be construed as a potential conflict of interest.

## Publisher's Note

All claims expressed in this article are solely those of the authors and do not necessarily represent those of their affiliated organizations, or those of the publisher, the editors and the reviewers. Any product that may be evaluated in this article, or claim that may be made by its manufacturer, is not guaranteed or endorsed by the publisher.

## Abbreviations

AAO: age at onset
ADL: activities of daily living
GWAS: genome-wide association study
HY: Hoehn and Yahr score
IVW: inverse variance weighted
LD: linkage disequilibrium
MR: Mendelian randomization
SEADL: the Schwab and England ADL
PD: Parkinson's disease
SNP: single nucleotide polymorphism
UPDRS: the Unified Parkinson's Disease Rating Scale.
