# Supplementary material for: Green Tea Intake and Parkinson's Disease Progression: A Mendelian Randomization Study
Source: Front Nutr. 2022 May 26;9:848223. doi: 10.3389/fnut.2022.848223 (PMC9199515; doi:10.3389/fnut.2022.848223)
Supplement: Supplementary file 1 [file Data_Sheet_1.PDF]

Supplementary Figure 1. Schematic analysis workflow.

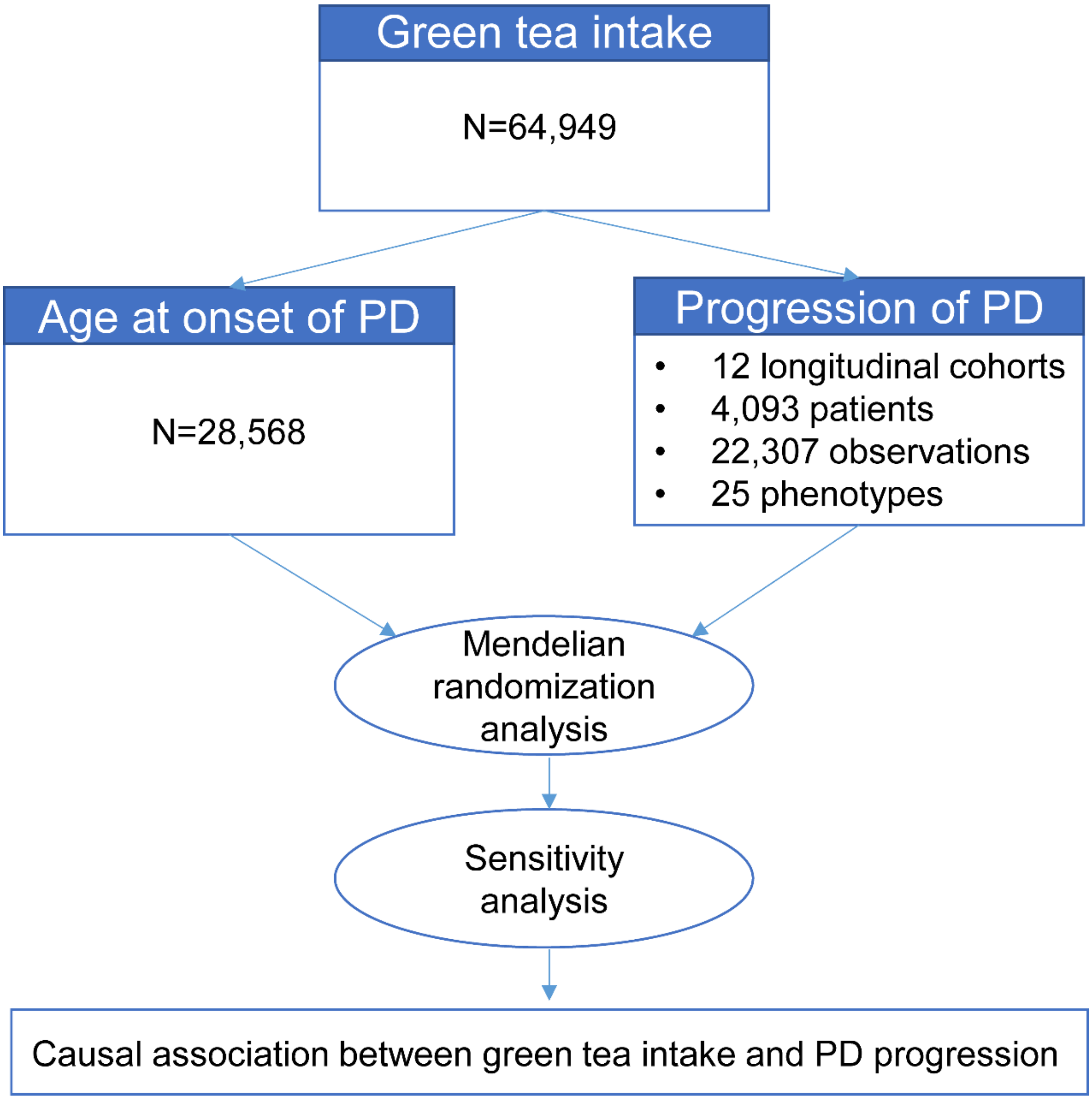

Supplementary Figure 2. Assumptions in Mendelian randomization analysis.

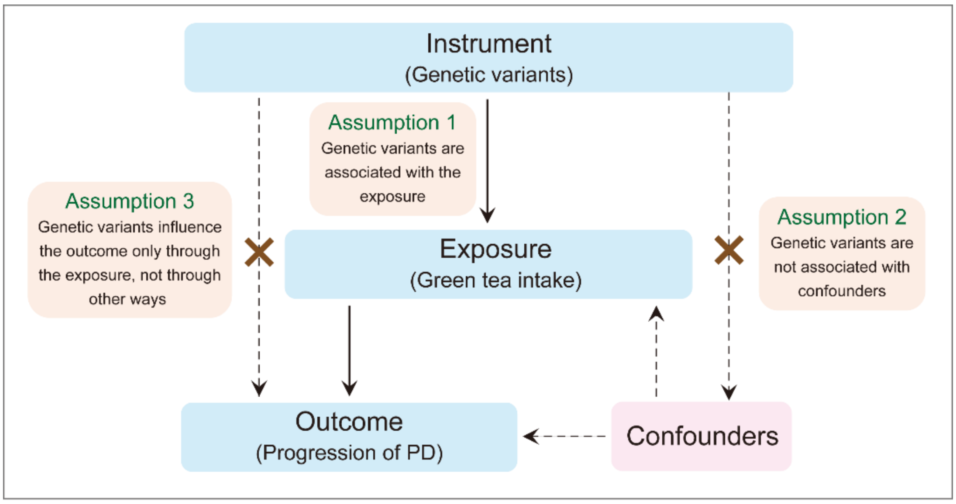

Broken lines represent potential pleiotropic or direct causal effects between variables that would violate Mendelian randomization assumptions. Assumption 1: Genetic variants are associated with the green tea intake; Assumption 2: Genetic variants are not associated with confounders; Assumption 3: Genetic variants influence the PD progression only through the exposure

Supplementary Figure 3. Mendelian randomization analysis results for green tea intake on risk of dementia at baseline.

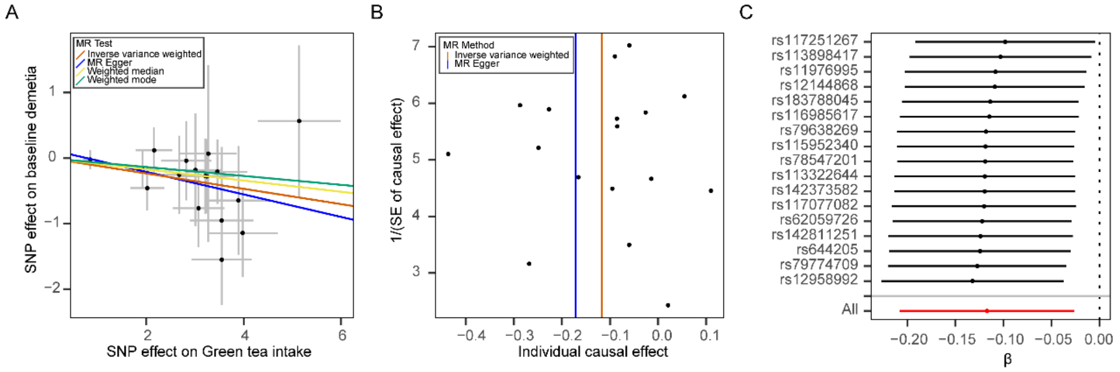

(A) Scatter plot of single nucleotide polymorphism (SNP) potential effects on green tea intake and dementia. The 95% CI for the effect size on green tea intake is shown as vertical lines, while the 95% CI for the effect size on dementia of PD is shown as horizontal lines. The slope of fitted lines represents the estimated MR effect per method. (**B)** Funnel plot for green tea intake shows the estimation using the inverse of the standard error of the causal estimate with each individual SNP as a tool. The vertical line represents the estimated causal effect obtained using IVW and MR-Egger methods. (**C**) Forest plot of the results of the leave-one-out sensitivity analysis, where each SNP in the instrument was iteratively removed from the instrument variables.

Supplementary Figure 4. Mendelian randomization analysis results for green tea intake on risk of depression at baseline.

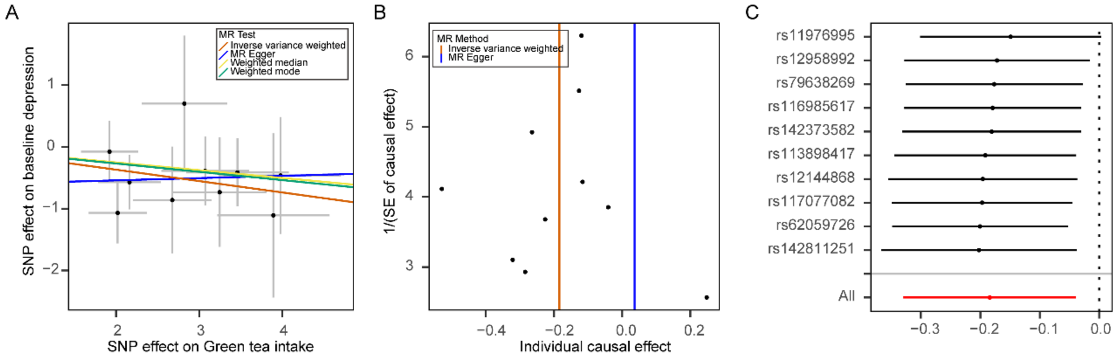

Supplementary Figure 5. Mendelian randomization analysis results for green tea intake on risk of hyposmia at baseline.

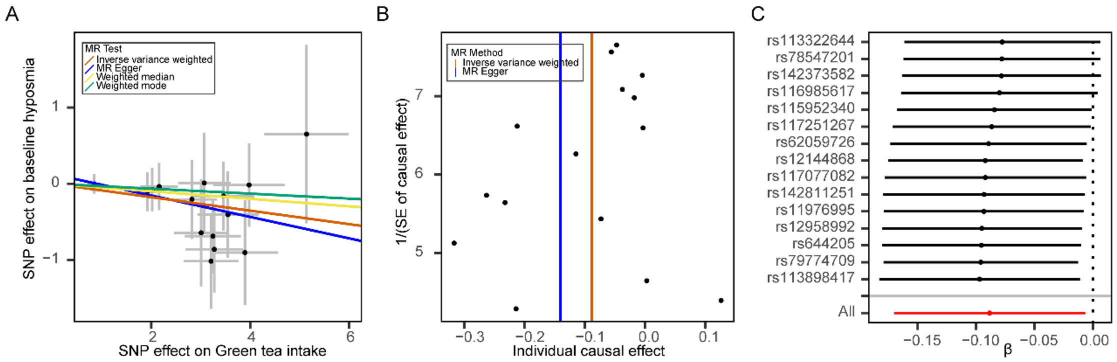

Supplementary Figure 6. Mendelian randomization analysis results for green tea intake on risk of insomnia at baseline.

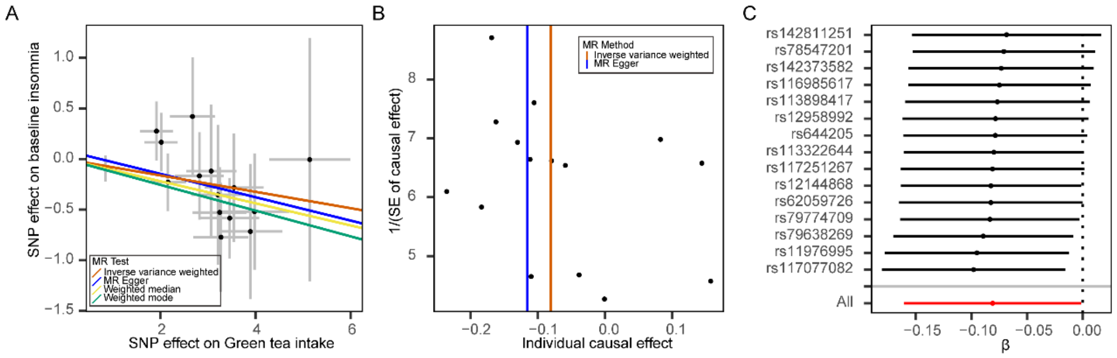

Supplementary Figure 7. Mendelian randomization analysis results for green tea intake on risk of progression to depression.

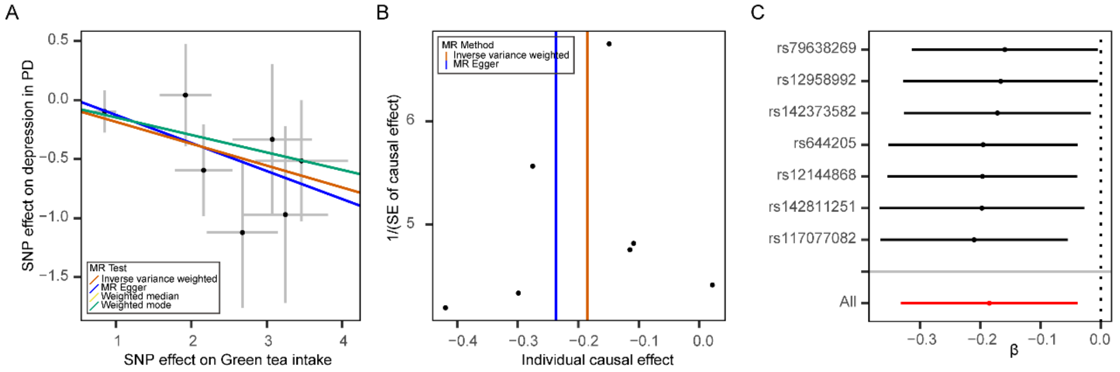

Supplementary Table 1. Phenotypes significantly associated with instrumental variables of green tea intake identified by PhenoScanner.

SNP	associated phenotype	P value	Pubmed ID
rs113322644	Otosclerosis	1.40E-08	UKBB
rs113898417	n.a.	n.a.	n.a.
rs115952340	n.a.	n.a.	n.a.
rs116985617	n.a.	n.a.	n.a.
rs117077082	n.a.	n.a.	n.a.
rs117251267	n.a.	n.a.	n.a.
rs11976995	n.a.	n.a.	n.a.
rs12144868	n.a.	n.a.	n.a.
rs12958992	n.a.	n.a.	n.a.
rs142373582	n.a.	n.a.	n.a.
rs142811251	Treatment with hyoscine butylbromide	1.84E-08	UKBB
rs144954030	n.a.	n.a.	n.a.
rs145313301	n.a.	n.a.	n.a.
rs183788045	n.a.	n.a.	n.a.
rs189140232	n.a.	n.a.	n.a.
rs62059726	n.a.	n.a.	n.a.
rs644205	Eosinophil count	2.49E-09	27863252
	Mean corpuscular volume	1.43E-08	27863252
	Sum eosinophil basophil counts	3.33E-08	27863252
	Relative age of first facial hair	3.72E-08	UKBB
rs78547201	n.a.	n.a.	n.a.
rs79638269	n.a.	n.a.	n.a.
rs79774709	n.a.	n.a.	n.a.

n.a., not available.
